# The emerging role of cardiovascular magnetic resonance in the evaluation of cardiac involvement in systemic sclerosis

**DOI:** 10.1515/rir-2024-0012

**Published:** 2024-07-15

**Authors:** Sophie I. Mavrogeni, Alessia Pepe

**Affiliations:** Exercise Physiology and Sport Medicine Clinic, Center for Adolescent Medicine and UNESCO Chair in Adolescent Health Care, First Department of Pediatrics, School of Medicine, National and Kapodistrian University of Athens, Aghia Sophia Children’s Hospital, 115 27 Athens, Greece; Onassis Cardiac Surgery Clinic, Athens, Greece; Institute of Radiology, Department of Medicine, University of Padua, Padua, Italy

**Keywords:** systemic sclerosis, cardiac fibrosis, cardiovascular magnetic resonance

## Abstract

Systemic sclerosis (SSc) is an autoimmune rheumatic disease, characterized by vascular, inflammatory and fibrotic alterations. Cardiac involvement is the « fatal tip of the iceberg» in SSc, as it leads to high morbidity/mortality. Cardiovascular imaging modalities play an important role in the early diagnosis and treatment assessment of cardiac involvement. Echocardiography is the corner stone for evaluation of cardiac involvement, providing information about function, wall motion, pulmonary pressure, pericardium and valvular disease. It is a low-cost modality, widely available, without radiation and with great experience among cardiologists. However, it is a window and operator dependent modality and cannot provide tissue characterization information, absolutely necessary for diagnosis and treatment of cardiac involvement in SSc. Cardiovascular magnetic resonance (CMR) can perform myocardial function and tissue characterization in the same examination without radiation, has excellent reproducibility and is window and operator independent. The great advantage of CMR is the capability to assess peri- myo-vascular inflammation, myocardial ischemia and presence of replacement and diffuse myocardial fibrosis in parallel with ventricular function assessment. The modified Lake Louise criteria including T2, native T1 mapping and extracellular volume fraction (ECV) has been recently used to diagnose inflammatory cardiomyopathy. According to expert recommendations, myocardial inflammation should be considered if at least 2 indices, one T2 and one T1 parameter are positive, whereas native T1 mapping and ECV assess diffuse fibrosis or oedema, even in the absence of late gadolinium enhancement (LGE). Moreover, transmural/subendocardial LGE following the distribution of coronary arteries and diffuse subendocardial fibrosis not related with epicardial coronary arteries are indicative of epicardial and micro-vascular coronary artery disease, respectively. To conclude, CMR can overcome the limitations of echocardiography by identifying acute/active or chronic myocardial inflammation/fibrosis, ischemia and myocardial infarction using classic and parametric indices in parallel with biventricular function assessment

## Introduction

Systemic sclerosis (SSc) is a chronic, autoimmune rheumatic disease, characterised by autoimmune reactivity, vascular dysfunction, inflammation and enhanced fibroblast activity leading to internal organ fibrosis, including the heart.^[1]^ Mortality in SSc is higher compared to other systemic rheumatic diseases.^[2]^ The 2010 survey from the European League Against Reumatism Scleroderma Trials and Research (EUSTAR) database estimated that 26% of SSc-related causes of death were due to cardiovascular (CV) causes (mainly heart failure and arrhytmias) and 29% of non-SSc-related causes of death were due to CV causes.^[3]^ Therefore, it is considered as the “fatal tip of the iceberg” in this disease.

Myocardial fibrosis either diffuse or localised may trigger various types of arrhythmias and also lead to heart failure (HF). It may be due to sustain or episodic myocardial inflammation and/or microcirculatory ischaemia.^[1,4]^ The better understanding of the pathophysiologic background may lead to effective treatment.^[4, 5, 6, 7]^

Echocardiography is the corner stone for the evaluation of CV involvement, providing information about function, wall motion, pulmonary pressure, pericardial status and valvular disease. It is a low-cost modality, widely available, without radiation and with great experience among cardiologists. However, it is a window and operator dependent modality and cannot provide tissue characterization information, absolutely necessary for diagnosis and treatment of cardiac involvement in SSc.^[8]^ The application of new echocardiographic techniques showed that SSc patients have a lower strain than healthy controls, indicating the presence of myocardial alterations involving both ventricles and atriums.^[9]^ However, echocardiography cannot provide details about the presence of myocardial inflammation/fibrosis, which can guide further treatment.^[8]^

## Role of Cardiovascular Magnetic Resonance

Cardiovascular magnetic resonance (CMR), a non-invasive, radiation free modality, has been successfully used to assess cardiac function, inflammation /fibrosis and myocardial ischemia in various myocardial diseases.^[7,8,10, 11, 12]^ CMR can perform:

### A) Evaluation of Cardiac Function

It is the gold standard for the noninvasive, non-contrast assessment of ventricular volumes and ejection fraction. It is of great value for the evaluation of the right ventricle, which is of special interest for SSc and is not always adequately imaged by echocardiography. CMR provides 3-dimensional images of the heart, which is also feasible with 3D echocardiography. However, in patients with HF CMR is more accurate than 3D echocardiography.^[13]^

### B) Assessment of Myocardial Inflammation

Myocardial inflammation is currently defined, using CMR, according to the Journal of the American College of Cardiology (JACC) Scientific Expert Panel provided consensus recommendations for an update of the Lake Louise diagnostic criteria (LLC) for myocardial inflammation in patients with suspected acute or active myocardial inflammation that include parametric mapping techniques.^[12]^ The authors proposed that CMR provides strong evidence for myocardial inflammation, with increasing specificity, if it demonstrates the combination of myocardial oedema with other CMR markers of inflammatory myocardial injury.

**Figure 1:**
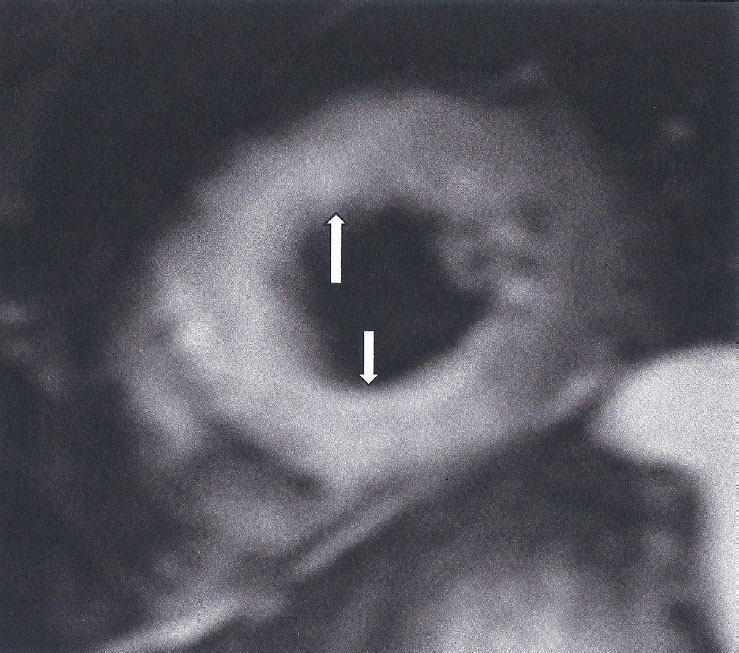
High myocardial signal (white area in STIRT2 images) indicative of myocardial oedema

This is based on at least one T2-based criterion (global or regional increase of myocardial T2 relaxation time or an increased signal intensity in T2-weighted CMR images), with at least one T1-based criterion (increased myocardial T1, extracellular volume, or late gadolinium enhancement). While having both a positive T2-based and a T1-based marker will increase specificity for diagnosing acute/active myocardial inflammation, having only one (*i.e*., T2-based or T1-based) marker may still support a diagnosis of acute myocardial inflammation in an appropriate clinical scenario, but with less specificity ^[12]^ (Figure 1).

### C) Assessment of Myocardial Fibrosis

#### Replacement (Focal) Fibrosis Using Late Gadolinium Enhanced Images (LGE)

LGE, taken 10-15 min after the use of paramagnetic contrast agent gadolinium, detect focal (replacement) myocardial fibrotic tissue (scar), if T2 weighted images are normal; it appears as a bright area in a background of suppressed, black myocardium.^[13]^ Replacement fibrosis can be either ischemic^[14,15]^ or inflammatory.^[16]^ The type of LGE and its clinical interpretation are presented in Table 1.

**Figure 2:**
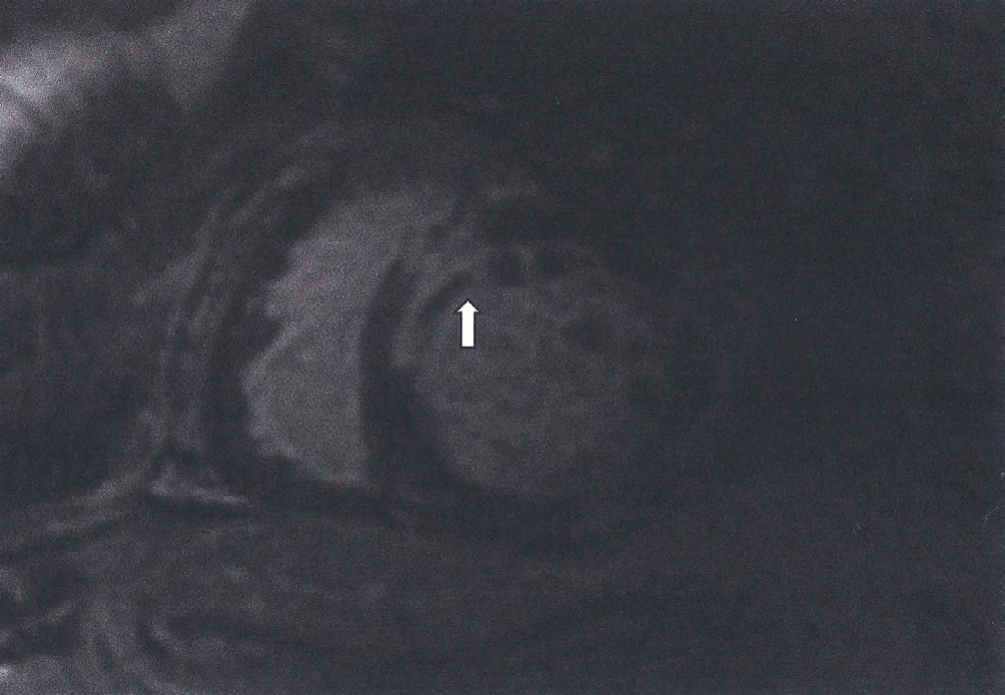
Subendocardal myocardial fibrosis (LGE) (white area of high signal) following the distribution of LAD in a SSc patient. The black area inside the white area represents microvascular obstruction

**Table 1:** The type of LGE and its clinical interpretation.

Type of LGE	Clinical interpretation
Subendocardial/transmural LGE, following the distribution of epicardial coronary arteries (Figure 2)	Epicardial coronary arteries disease
Subepicardial/intramyocardial (Figure 3)	Myocardial inflammation
Diffuse or small subendocardial LGE not following the distribution of epicardial coronary arteries or with normal epicardial coronary arteries (Figures 4, 5)	Micro-vascular coronary artery disease

**Figure 3:**
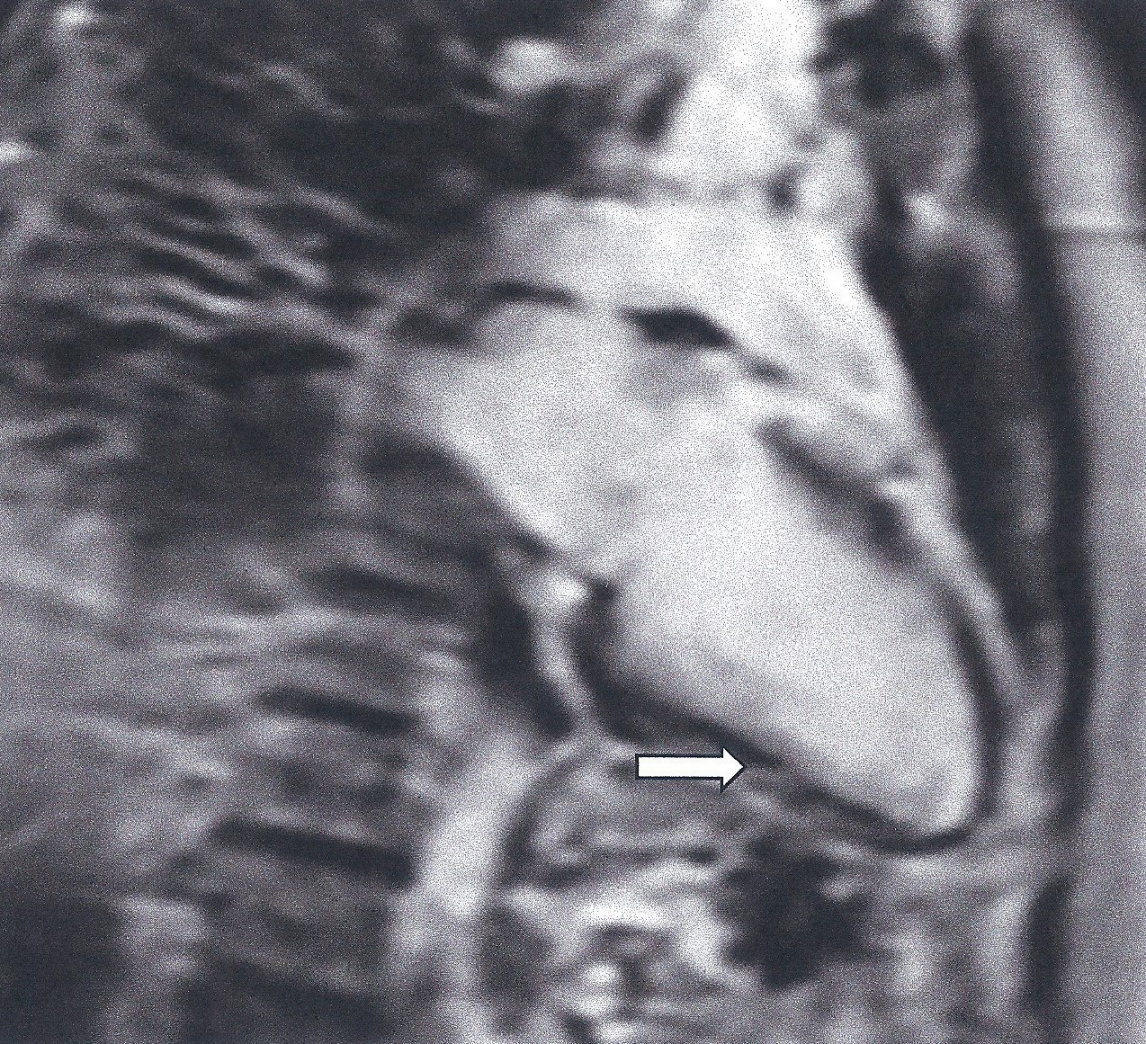
Subepicardial fibrosis (LGE) (spotty, white areas of high signal) in the inferior wall of LV, due to myocardial inflammation in a SSc patient

**Figure 4:**
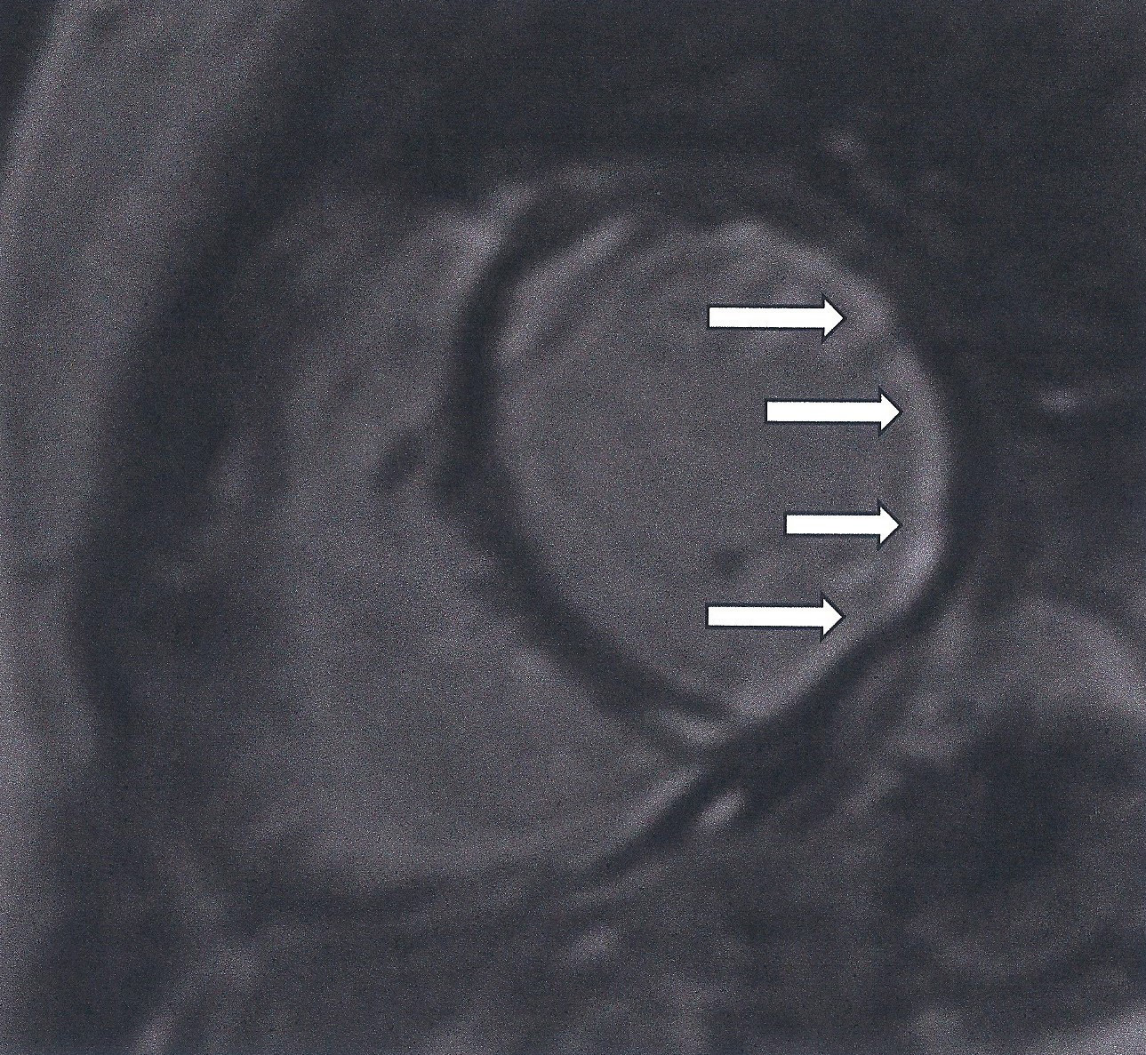
Diffuse subendocardial fibrosis (LGE) (white area of high signal), due to microvascular coronary artery disease in a patient with SSc

**Figure 5:**
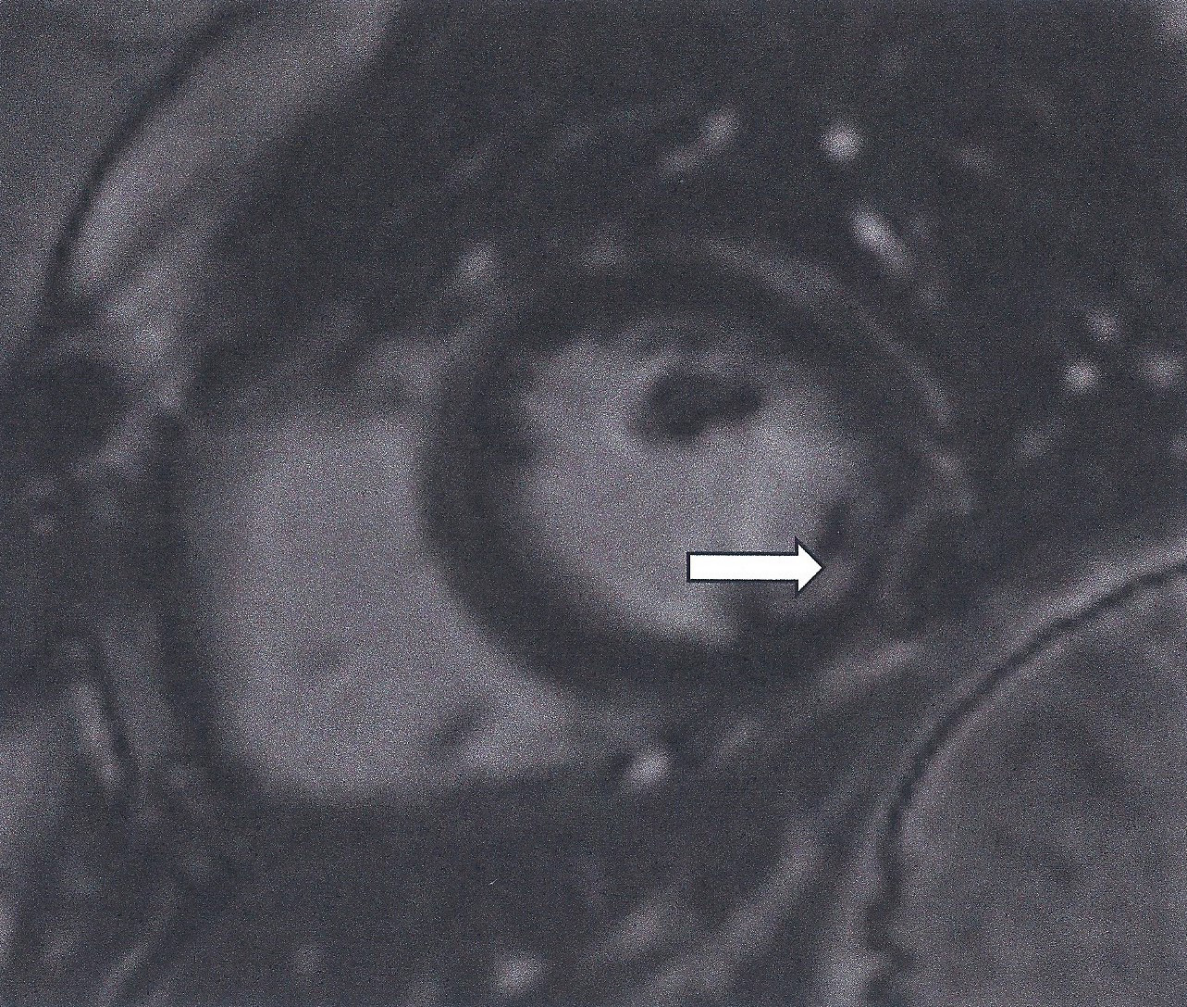
Small subendocardial fibrosis (LGE) (white area of high signal) in the lateral wall of a patient with SSc and normal epicardial coronary arteries

#### Diffuse Myocardial Fibrosis Using Native T1 Mapping and Extracellular Volume Index (ECV)

Although LGE has been validated as the technique of choice for the detection of replacement (focal) fibrosis, it has inherent disadvantages for the assessment of diffuse myocardial fibrosis, because it is based on the signal intensity differences between scarred and normal myocardium to generate image contrast. Since a normal myocardial reference value is required for the LGE images, this approach is unable to detect diffuse myocardial fibrosis, because there is no clear distinction between fibrotic tissue and normal myocardium, commonly found in SSc.^[17,18]^ To overcome this limitation, parametric (mapping) imaging was generated including T1, T2 mapping and ECV. Compared to LGE, native T1 mapping enables the identification of diffuse myocardial fibrosis, which is otherwise undetectable by the currently used circulating biomarkers. Furthermore, it has an excellent correlation with histology.^[19]^ Additionally, native T1 mapping can be measured in patients with severely reduced glomerular filtration rate (GFR) or chronic renal failure, because the application of contrast agent is not needed for the generation of these images.^[20]^ However, native T1 mapping values are strongly dependent on the local field strength, the vendor, and the sequence used. Therefore, a local reference range should be used. Furthermore, a segmental approach is suggested to increase the sensitivity.^[21]^

Contrast-enhanced T1 mapping is used for ECV calculation in combination with native T1 mapping. Standard gadolinium-based contrast agents are distributed throughout the extracellular space and shorten T1 relaxation times of the myocardium proportional to the local concentration of gadolinium. Areas of positive LGE will therefore exhibit shorter T1 relaxation times, in particular after contrast administration. The haematocrit represents the cellular fraction of blood. Estimation of ECV, which represents the interstitium and extracellular matrix, requires measurement of myocardial and blood T1 before and after administration of contrast agents as well as the patient’s haematocrit value according to the formula:

$$\begin{array}{c} ECV=(1-hematocrit)\frac{\left(1/T1myopostamtrast-1/T1myoprecontrast\right)}{\left(1/T1bloodpostcontrast-1/T1bloodprecontrast\right)} \end{array}$$

Normal ECV values of 25.3 ± 3.5% have been reported in healthy individuals at 1.5T.^[20,21]^ Apart from amyloid, an increased ECV is most often due to excessive collagen deposition as in SSc^[22]^ and therefore represents a more robust measure of myocardial fibrosis. Low ECV values occur in thrombus and fat/Lipomatous metaplasia. ECV can be calculated either from myocardial regions-of-interest or visualized on ECV maps.^[23]^ ECV represents a physiological parameter that it seems more reproducible among different field strengths, vendors and acquisition techniques than both native and post-contrast T1.^[24]^ Thus, for ECV, reference ranges from the literature using the same CMR system and same pulse sequence may be acceptable.^[24]^ Moreover, ECV measures also exhibit better agreement with histological measures of the collagen volume fraction than isolated post-contrast T1.^[25]^ CMR parametric (mapping) indices and their clinical interpretation are presented in Table 2.

**Table 2:** CMR parametric (mapping) indices and their clinical interpretation.

CMR parametric (mapping) indices	Clinical interpretation
Increased values of T2 mapping (oedema index)	Acute/active myocardial disease
Both native T1 mapping and ECV are influenced by oedema	They considered as indices of myocardial oedema in SSc patients with increased T2 mapping values
Increased values of T1 mapping and ECV, with normal T2 mapping	They are indicative of diffuse myocardial fibrosis, even in the absence of LGE

CMR, cardiovascular magnetic resonance; ECV, extracellular volume index; LGE, late gadolinium enhanced T1-W images.

### D) Assessment of Myocardial Ischemia in SSc Using CMR

Stress CMR is the ideal tool to assess the presence of myocardial ischemia. Vasodilator agents, such as dypiridamle, adenosine or regadenoson, can be used in parallel with paramagnetic agents and reveal the presence of ischemic areas. If the ischemic area follows the distribution of epicardial coronary arteries, is the result of epicardial coronary artery disease (ECAD). If there is a diffuse subendocardial perfusion defect, it reflects the presence of microvascular coronary artery disease (MCAD).^[26,27]^ Myocardial stress perfusion defects can be detected in SSc using pharmacological stress CMR perfusion. These defects are independent from traditional risk factors or associated comorbidities and considered as representing a hallmark of myocardial involvement in SSc.^[28]^ The CMR patterns of myocardial ischemia and their clinical interpretation are presented in Table 3.

**Table 3:** The CMR patterns of myocardial ischemia and their clinical interpretation.

Distribution of ischemia	Type of coronary artery disease
Perfusion defect following the distribution of epicardial coronary arteries	Epicardial coronary artery disease
Perfusion defect not following the distribution of epicardial coronary arteries	Micro-vascular coronary artery disease representing the hallmark of SSc^[25]^

SSc, systemic sclerosis.

## Conclusions

Cardiac involvement represents the “tip of the iceberg” in SSc and leads in increased mortality/morbidity. The detection of a myocardial inflammation / ischemia / fibrosis patterns can identify different aspects of cardiac pathophysiology of SSc.

Echocardiography using the new techniques can potentially detect some early myocardial alterations, but it cannot distinguish acute/active inflammation from chronic fibrotic process. In contrast, CMR is the only imaging modality without radiation that can assess myocardial disease acuity, presence of ECAD or MCAD, type of myocardial fibrosis (ischemic/nonischemic) and further guide therapeutic decision making.
